# Anti-Glutamic Acid Decarboxylase Antibody-Associated Ataxia as an Extrahepatic Autoimmune Manifestation of Hepatitis C Infection: A Case Report

**DOI:** 10.1155/2011/975152

**Published:** 2011-07-10

**Authors:** Amer Awad, Olaf Stüve, Marlyn Mayo, Rafeed Alkawadri, Bachir Estephan

**Affiliations:** ^1^Baton Rouge Neurology Associates, Baton Rouge General Medical Center, Baton Rouge, LA, USA; ^2^Department of Neurology, The University of Texas Southwestern Medical Center, Dallas, TX, USA; ^3^Neurology Section, VA North Texas Health Care Systems, Dallas, TX, USA; ^4^Department of Internal Medicine-Digestive and Liver Diseases, The University of Texas Southwestern Medical Center, Dallas, TX, USA; ^5^Neurological Institute, Cleveland Clinic Foundation, Cleveland, OH, USA; ^6^Department of Neurology, University of Kansas Medical Center, Kansas, KS, USA

## Abstract

Extrahepatic immunological manifestations of hepatitis C virus (HCV) are well described. In addition, antiglutamic acid decarboxylase (GAD) antibody-associated cerebellar ataxia is well-established entity. However, there have been no reports in the literature of anti-GAD antibody-associated ataxia as an extrahepatic manifestation of HCV infection. We report the case of a young woman with chronic hepatitis C virus and multiple extrahepatic autoimmune diseases including Sjögren syndrome and pernicious anemia who presented with subacute midline cerebellar syndrome and was found to have positive antiglutamic acid decarboxylase (GAD) antibody in the serum and cerebrospinal fluid. An extensive diagnostic workup to rule out neoplastic growths was negative, suggesting the diagnosis of nonparaneoplastic antiglutamic acid decarboxylase antibody-associated cerebellar ataxia as an additional extrahepatic manifestation of hepatitis C virus infection. The patient failed to respond to high-dose steroids and intravenous immunoglobulin. Treatment with the monoclonal antibody rituximab stabilized the disease. We postulate that anti-GAD associated ataxia could be an extrahepatic manifestation of HCV infection.

## 1. Introduction

Hepatitis C virus (HCV) is commonly associated with autoimmune diseases as extrahepatic manifestations (EHM) [1] The most important autoimmune diseases associated with HCV are mixed essential cryoglobulinemia (MEC) [2] and Sjögren syndrome (SS) [3]. Other autoimmune diseases have been described in patients with HCV, but the association has not been well documented. These autoimmune diseases include HCV-associated arthritis [4], systemic lupus erythematosus [5], polyarteritis nodosa [6], antiphospholipid antibody syndrome [7], inflammatory myopathies [8], sarcoidosis [9], autoimmune thyroid disease [10], autoimmune glomerulonephritis [11], skin vasculitis [12], and autoimmune thrombocytopenia [13].

The pathogenesis of these EHM is still not fully understood, although most studies suggest that the presence of MEC, particular lymphotropism of the virus, molecular mimicry, and non-MEC autoimmune phenomena constitute the major pathogenic factors [14]. 

To our knowledge, there have been no previous reports of antiglutamic acid decarboxylase (GAD) antibody-associated cerebellar ataxia as an extrahepatic manifestation of chronic HCV infection. We report here a young woman with chronic HCV infection, who presented with subacute midline (vermis) cerebellar syndrome and tested positive for anti-GAD antibodies in the serum and the cerebrospinal fluid (CSF). We postulate that the patient has a postinfectious or parainfectious autoimmune disease caused by antibodies directed against the neuronal antigen GAD 65.

## 2. Case Report

The patient is a 48-year-old African American woman with past medical history significant for HCV secondary to blood transfusion, SS, pernicious anemia, and obesity status postbariatric surgery. She presented to the neurology clinic with history of subacute onset gait ataxia, intermittent vertigo, diplopia, oscillopsia, dysarthria, and dysphagia. The patient was initially treated with high-dose intravenous methylprednisolone (IVMP) followed by high-dose oral prednisone with modest response as her ataxia continued to progress. Based on the assumption that her symptoms were secondary to central nervous system (CNS) involvement of SS, she was treated with rituximab with no significant clinical improvement though it stabilized the disease. The patient also did not respond to intravenous immunoglobulin (IVIG) treatment. No history of alcohol use or malnutrition. Her neurological examination revealed hypometric saccades, mild dysarthria, truncal ataxia, and gait ataxia without limb ataxia. A motor exam was unremarkable, and sensory exam was positive for decreased vibratory sensation distally. An extensive workup was initiated. Abnormal results include HCV viral load 193,000 copies per mL, HCV genotype I, liver biopsy (stage I HCV disease), antinuclear antibody (Ab) (ANA) positive, SS-A positive (1 : 230), antiparietal cell Ab positive, anti-intrinsic factor (IF) Ab positive, small M spike on serum protein electrophoresis (SPEP) with normal 24-hour urine protein electrophoresis (UPEP), cerebrospinal fluid (CSF) oligoclonal bands (OCBs) positive with normal cell count and protein, and increased uptake on the right submandibular gland on positron emission tomography (PET). Pertinent negative/normal results include vitamin B12 (on supplements), folic acid (on supplements), vitamin B1 (thiamine on supplements), vitamin B6, vitamin E, lactate, pyruvate, thyroid stimulating hormone (TSH), copper, ceruloplasmin, urine heavy metals, human immunodeficiency virus (HIV), rapid plasma regain (RPR), SS-B, rheumatoid factor (RF), antineutrophilic cytoplasmic Ab (ANCA), antiphospholipid Ab (a PL), angiotensin-converting enzyme (ACE), antiendomysial Ab, erythrocyte sedimentation rate (ESR), C-reactive protein (CRP), C3, C4, cryoglobulins, paraneoplastic panel, anti-amphiphysin antibody, brain and spine magnetic resonance imaging (MRI) with contrast, bone scan, bone marrow biopsy, mammogram, Pap smear, computerized tomography (CT) of the chest, abdomen, and pelvis. The most remarkable finding was a positive anti-GAD 65 Ab in the serum (titer 113.65 units per mL) and in the CSF (titer 29.4 nmole/L with a normal range of <0.02 nmole/L) with positive OCBs despite treatment with steroids, IVIG, and Rituximab. The patient had numerous fasting blood sugars (FBS) done to exclude late onset diabetes. All the FBS values were within normal limits (80–95 mg/dL). 

Antiviral therapy with interferon alpha (IFN*α*) and ribavirin was discussed with the hepatologist, but the patient was considered a poor candidate due to the possibility of exacerbation of a secondary autoimmune disease. Since the patient's cerebellar syndrome was stabilized by rituximab, we continued maintenance therapy with close monitoring of her neurological status.

## 3. Discussion

The patient presented with symptoms and signs consistent with cerebellar vermis syndrome including hypometric saccades, truncal, and gait ataxia without limb ataxia [15, 16]. Thiamine and copper deficiency as potential causes of ataxia, which are common after Bariatric surgery [17, 18], were excluded by normal serum levels. The patient was on vitamin B12 and folic acid supplements. Brain and spine MRI excluded structural causes of ataxia. The thorough workup excluded other potential causes of ataxia including vitamin E deficiency [19], heavy metal toxicity [20, 21], celiac disease-associated ataxia [22]. Finally, numerous fasting blood sugar samples excluded late onset insulin-dependent diabetes mellitus. 

The most striking abnormality was the finding of positive anti-GAD 65 Abs in the serum and the CSF. The positivity in the CSF confirms its pathogenic role in the patient's ataxia. Anti-GAD 65 Ab has been associated with a myriad of disorders including insulin-dependent diabetes mellitus (IDDM) [23], stiff person syndrome (SPS) [24], limbic encephalitis [25], opsoclonus-myoclonus syndrome [26], epilepsy [27], and ataxia [27, 28]. The positivity for anti-GAD dictated a thorough neoplastic workup because anti-GAD antibody is often associated with neoplastic growth in the context of a paraneoplastic neurological disorder. This is especially true if anti-amphiphysin antibodies are also present [29]. A comprehensive neoplastic workup was negative, and a paraneoplastic antibody panel was negative as well. The clinical picture in parallel with the lab findings suggests the diagnosis of nonparaneoplastic anti-GAD Ab-associated ataxia. Nevertheless, the continuous and long-term search for occult neoplasms is required to confirm the nonparaneoplastic origin since neurological paraneoplastic disorders can precede the diagnosis of occult malignancies by a long period of time.

While ataxia associated with anti-GAD antibodies is a well-described entity, this patient is unusual as her underlying chronic medical condition is a viral infection of the liver, namely, HCV. A thorough literature review did not uncover previous reports of such an association. We propose that the anti-GAD ataxia in this patient is an extrahepatic autoimmune manifestation of HCV. The association might be incidental, but there are 2 factors that make the association more than just incidental. The first one is the extreme rarity of anti-GAD ataxia making the mathematical possibility of both occurring in the same patient very highly unlikely. The second factor, and the more important reason is the finding of remarkable molecular mimicry between HCV and GAD 65 [30].

With regard to the etiology of this clinical presentation, we hypothesize that this patient is suffering from a para-infectious autoimmune disease caused by of molecular mimicry. Not unlike in a paraneoplastic syndrome, it is conceivable that a neuronal antigen-like determinant is ectopically expressed. Only in this case, it would be expressed not by a malignant cell, but instead either by HCV, or by a host cell infected with HCV. Consequently, this antigen is recognized as “foreign”. Initially, the viral neuronal-antigen-like determinant is recognized at the site of inoculation by cells of the innate immune system, which then present linear epitopes to T cells. These T cells, once activated, in turn cross-activates antigen-specific B cells. Such activated autoreactive T cells and B cells readily entering the CNS during the course of routine immune surveillance [31]. Incidentally, these cells may encounter a neuronal antigen in the cerebellum that closely resembles the viral neuronal-antigen-like determinant they were originally primed against. Following reactivation by CNS intrinsic antigen presenting cells, these T cells and B cells now initiate and autoimmune disease, which includes the recruitment of nonantigen specific immune cells from the blood that amplify the inflammation. 

It would be extremely difficult to test our hypothesis in a human patient. In animal models, the cascade of events described above was successfully demonstrated experimentally by transgenic insertion of a lymphocytic choriomeningitis virus (LCMV) antigen in murine oligodendrocytes. Following intraperitoneal inoculation of these animals with LCMV, the infection was cleared at the entry site. However, 7–14 days later, the inflammation of the CNS was detected, and the experimental animals displayed functional clinical deficits [32].

Treatment of anti-GAD syndrome is not well established yet. However, favorable responses to steroids, IVIG, and plasma exchange were reported in the literature [33–35]. Rituximab was shown to be safe and helpful in treatment of EHM of HCV [36, 37]. More importantly, antiviral treatment of HCV can improve EHM [38] but may worsen the course of autoimmune disorders associated with HCV [39]. 

## 4. Conclusion

Our case of HCV-associated anti-GAD ataxia should help alert neurologists and other physicians treating patients with HCV to the presence of this entity. Patients with HCV and neurological symptoms should be evaluated for neuroimmunological disorders by running a comprehensive immune workup including testing for anti-GAD Ab. Early diagnosis is crucial to stabilize the disease and prevent its progression since reversing neuropathology is currently not feasible. Further studies are crucial to study this association and the exact immunopathogenesis behind it. 
